# Diagnostic Utility of Bronchoalveolar Lavage in Patients with Acute Leukemia under Broad-Spectrum Anti-Infective Treatment

**DOI:** 10.3390/cancers14112773

**Published:** 2022-06-02

**Authors:** Susanne Ghandili, Philipp H. von Kroge, Marcel Simon, Frank O. Henes, Holger Rohde, Armin Hoffmann, Nick Benjamin Lindeman, Carsten Bokemeyer, Walter Fiedler, Franziska Modemann

**Affiliations:** 1 Department of Oncology, Hematology and Bone Marrow Transplantation with Section Pneumology, University Cancer Center Hamburg, University Medical Center Hamburg-Eppendorf, Martinistraße 52, 20246 Hamburg, Germany; s.ghandili@uke.de (S.G.); m.simon@uke.de (M.S.); nickben97@aol.com (N.B.L.); c.bokemeyer@uke.de (C.B.); fiedler@uke.de (W.F.); 2 Department of General, Visceral and Thoracic Surgery, University Medical Center Hamburg-Eppendorf, Martinistraße 52, 20246 Hamburg, Germany; p.von-kroge@uke.de; 3 Department for Diagnostic and Interventional Radiology and Nuclear Medicine, University Medical Center Hamburg-Eppendorf, Martinistraße 52, 20246 Hamburg, Germany; f.henes@uke.de; 4 The Institute of Medical Microbiology, Virology and Hygiene, University Medical Center Hamburg-Eppendorf, Martinistraße 52, 20246 Hamburg, Germany; rohde@uke.de (H.R.); ar.hoffmann@uke.de (A.H.); 5 Mildred Scheel Cancer Career Center, University Cancer Center Hamburg, University Medical Center Hamburg-Eppendorf, Martinistraße 52, 20246 Hamburg, Germany

**Keywords:** acute leukemia, pneumonia, flexible bronchoscopy, bronchoalveolar lavage

## Abstract

**Simple Summary:**

Infections of bacterial, viral, or fungal origin pose a major threat to patients with acute leukemia. Empiric antibiotic and antifungal treatment is a commonly used approach in leukemia patients with febrile neutropenia. To investigate the utility of flexible bronchoscopy (FB) with bronchoalveolar lavage (BAL) in acute leukemia (AL) patients with pneumonia who were already treated with broad-spectrum antibiotics and antifungal agents, we investigated bronchoalveolar samples of 88 patients. Our results indicate that despite broad-spectrum anti-infective treatment, in approximately half of all patients, pathogens could still be isolated in bronchoalveolar samples. Nevertheless, the detection of pathogens does not lead to frequent changes in anti-infective treatments, with most changes performed in patients with herpes simplex and influenza virus detection, and these pathogens might also be detected in less invasive examinations. The need for FB with BAL in patients with AL who are already receiving broad-spectrum empiric anti-infective treatment should therefore be weighed carefully.

**Abstract:**

Despite therapeutic advances in the prevention and treatment of febrile neutropenia, acute leukemia (AL) patients still have considerable febrile neutropenia-related mortality. However, the diagnostic yield of flexible bronchoscopy (FB) and bronchoalveolar lavage (BAL) in acute leukemia patients is unclear. In this retrospective single-center study, we analyzed 88 BAL samples of patients with acute leukemia and pulmonary infiltrates in spite of treatment with broad-spectrum anti-infective agents. The aim was to investigate the impact of FB with BAL on detecting causative organisms, which would result in a change in treatment regimens. The median age was 59 years, and 86% had acute myeloid leukemia. In 47%, pathogens were detectable in BAL fluid (pathogen bacteria, viruses, and fungi in 2, 15, and 18%, respectively), with *Aspergillus fumigatus* detected most frequently. BAL-guided anti-infective therapy changes were performed in 15%. The detection of herpes simplex and influenza viruses were the main reasons for treatment changes. Despite broad-spectrum anti-infective treatment, in approximately half of all patients, pathogens could still be isolated in BAL samples. However, consecutive changes in anti-infective treatment were considerably less frequent, with most changes performed in patients with *Herpes simplex virus* and *Influenza A* detection. The need for FB with BAL in patients with AL receiving broad-spectrum empiric anti-infective treatment should therefore be weighed carefully.

## 1. Introduction

Infections of bacterial, viral, or fungal origin pose a major threat to patients with acute leukemia (AL), both at the initial diagnosis of AL and particularly during intensive chemotherapy due to prolonged, profound neutropenia [1]. Therefore, severe infections and febrile neutropenia (FN) are among the most common complications in these patients. AL patients show the highest FN-related in-hospital mortality rates (14.3%) among all cancer types [2,3,4,5]. Thus, early detection of causative pathogens and their effective treatment would be crucial. However, frequently, empiric anti-infective treatment is necessary.

In general, there are different approaches to treat leukemia patients with FN or signs of pulmonary infection: (a) Empiric (treatment without clear focus or detection of a pathogen), (b) pre-emptive (treatment with clear focus or detection of a pathogen), or (c) targeted (treatment after resistance testing of pathogens) approaches are used [6]. An advantage of the empiric anti-infective treatment is the rapid administration of anti-infective agents with a prompt reaction regarding the deterioration of patients. Additionally, not every patient is administered invasive diagnostics, and many patients respond to empiric anti-infective treatment. On the other hand, pre-emptive approaches might be less cost-intensive but have the risk of false-negative results, for example, in low-dose chest computed tomography (LDCT) or flexible bronchoscopy (FB), undersupplying patients with anti-infective agents. However, despite regular microbiological examinations of blood, urine, and stool samples, including extensive testing for opportunistic pathogens by polymerase chain reaction (PCR), the detection of pathogenic germs often remains unsuccessful [4,7,8]. Pathogens can be detected in only 20–25% of cancer patients as the cause of FN. In another 20–25%, a clinical infection focus may be the cause for FN without detectable pathogens, whereas in the remaining 50%, no pathogen or infection focus can be found [4,7,8].

By adding LDCT in cases of ongoing FN despite empiric anti-infective treatment, pneumonia can be detected in 50% to 95% of FN-patients in whom no infection focus was previously detectable [9,10,11]. According to the current ESMO (European Society for Medical Oncology) clinical practice guidelines on the management of FN, patients with acute myeloid leukemia (AML) during induction therapy are particularly at risk for invasive fungal infections. Following these guidelines, flexible bronchoscopy (FB) with bronchoalveolar lavage (BAL) is recommended if any pulmonary infiltrate is detected by chest computed tomography (CT) [1]. Even though prospective data regarding the diagnostic utility of BAL in immunocompromised patients, including patients with hematological malignancies and those undergoing allogeneic stem cell transplantation, are existing, the diagnostic utility of BAL is still discussed controversially, especially if patients already receive prophylactic or therapeutic antifungals [1,12,13,14]. Moreover, less is known about the utility of FB and BAL and the possible impact on anti-infective treatment in patients with AL in the setting of ongoing FN or suspected infection despite broad-spectrum anti-infective treatment.

Here, we report on a single-center retrospective analysis with the aim to investigate the utility of FB and BAL and possible clinical implications in patients with AL and pulmonary infiltrates who are already empirically treated with broad-spectrum anti-infective agents.

## 2. Materials and Methods

### 2.1. Study Design and Population

**Figure 1:**
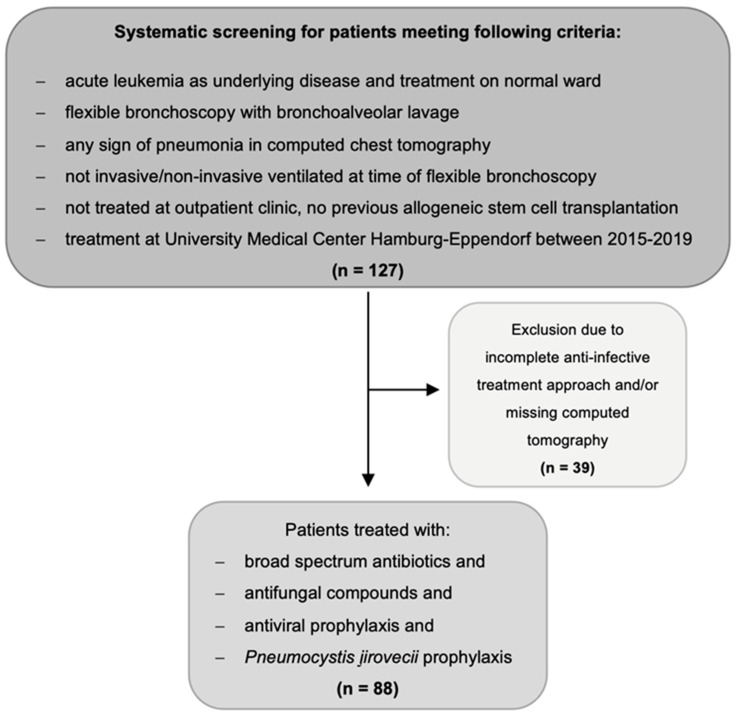
Flow chart of study design and population.

**Figure 2:**
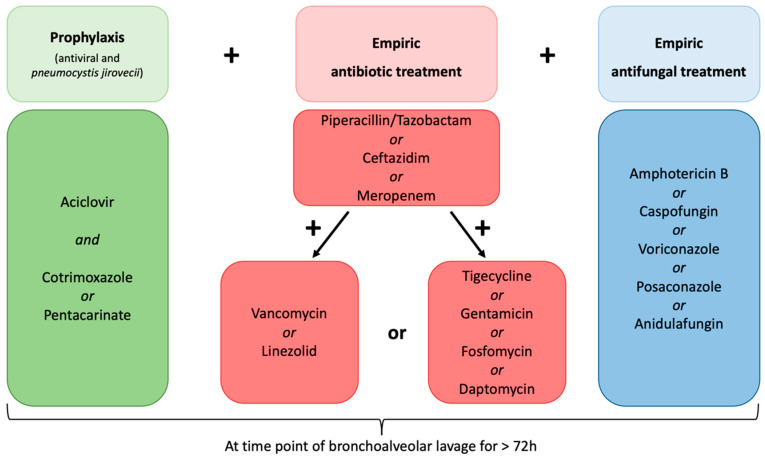
Hospital-based anti-infective treatment at time point of flexible bronchoscopy with bronchoalveolar lavage in each patient. All patients included in this study received empiric antibiotic and antifungal treatment for at least 72 h prior to bronchoscopy. The choice of empiric gram-positive targeted therapy and/or application of last-resort antibiotics and the choice of which antifungal agent was administered was at the discretion of the physician.

In this retrospective single-center study, patients were included if they were treated at the Department of Oncology and Hematology at the University Medical Center Hamburg-Eppendorf, Germany, between January 2015 and December 2019 and met the following inclusion criteria (Figure 1):

1. Aged ≥ 18 years.
2. Diagnosed with either AML or acute lymphoblastic leukemia (ALL).
3. Diagnosed with multi-drug resistant fever or suspected infection defined as an occurrence under >three days of empiric broad-spectrum antibiotic and empiric antifungal treatment, antiviral prophylaxis, and prophylaxis against *Pneumocystis jirovecii* (Figure 2).
4. Underwent CT scan of the chest showing pulmonary infiltrates within five days prior to FB with BAL.
5. Underwent procedure of FB with BAL.
6. No previous allogeneic stem cell transplantation.

Since the treatment of AL includes several cycles of chemotherapy, some patients have been enrolled more than once. Patients treated in an out-patient setting or at an intensive care unit at the time point of FB were excluded, as well as patients under non-invasive ventilation or those intubated at the time of bronchoscopy.

Subfebrile temperature was defined as body temperature ≥37.8 °C Fever was defined as body temperature ≥38.3 °C. Neutropenia was defined as neutrophile count <500/µL according to grade IV neutropenia defined by Common Toxicity Criteria for Adverse Events (CTCAE) v5.0 grading [15]. High-risk neutropenia was defined as neutrophile count ≥ 7 days < 500/µL.

### 2.2. Clinical Data Collection

Clinical data regarding treatment and disease characterization were collected from the patient’s electronic medical records. Laboratory investigations, LDCT, and FB with BAL were obtained during the clinical routine.

Retrospective data collection was performed in accordance with local legal requirements and was reviewed and approved by the Ethics Committee of the Medical Council of Hamburg (vote number PV7335). Informed consent was waived by the ethics committee since only pseudonymous data were analyzed and published.

### 2.3. Procedure of Flexible Bronchoscopy and Bronchoalveolar Lavage

FB with BAL was performed in a standardized manner by an experienced pulmonologist. In the preparation of all procedures, an LDCT was reviewed to assess the characteristics and location of lung parenchymal changes. Coagulation studies and platelet counts were obtained to assess the risk of bleeding. In the case platelet count was below 20,000 per µL, a platelet transfusion was administered prior to bronchoscopy. Intravenous sedation for the procedure was provided according to patient preference. It was achieved using a bolus application of 2 mg of midazolam and repetitive bolus applications of 10 to 20 mg of propofol every two to three minutes. Topical anesthesia was applied to the laryngeal, tracheal, and bronchial mucosa using 5 mL of lidocaine 0.8% applied through the working channel of the bronchoscope in aliquots of 1 mL. A flexible video bronchoscope (Olympus, Tokyo, Japan) passed through the mouth was used for the procedures. After inspection of the tracheobronchial tree, the bronchoscope was advanced into the appropriate airway. BAL was then performed using normal saline at room temperature, being instilled in aliquots of 20 mL and then aspirated. After completion of the procedure, BAL fluid was immediately sent to the laboratory for microbiological testing.

### 2.4. Microbiological Tests

**Figure 3:**
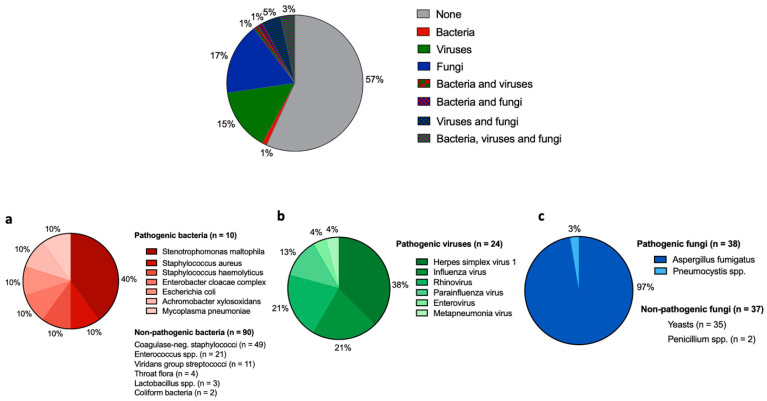
Detection of pathogenic bacteria, viruses, or fungi in bronchoalveolar fluid and distribution of all detected clinically relevant pathogens by showing rates of detection of bacteria, virus, and fungi, or a combination of them in all 88 cases. (**a**) Rate of pathogenic bacteria detected in total of all BAL fluids. Non-pathogenic bacteria are named below but not in the figure. (**b**) Rate of pathogenic viruses detected in total of all BAL fluids. Non-pathogenic viruses were not detected. (**c**) Rate of pathogenic fungi detected in total of all BAL fluids. Non-pathogenic bacteria are named below but not in the figure.

Microbiological tests in each patient included microscopic and cultural examination for bacteria, mycobacteria and fungi, Aspergillus galactomannan, *Pneumocystis jirovecii* PCR, *Cytomegalovirus* PCR, and *Herpes simplex virus* (HSV) PCR. Furthermore, multiplex PCR for common respiratory viruses was performed including adenoviruses, bocaviruses, coronaviruses (OC43, HKU1, NL63, 229E), enteroviruses, human metapneumovirus, *Influenza A and B*, *Parainfluenza 1-4*, *Respiratory syncytial virus*, and *Rhinovirus*. Multiplex PCR for *Mucor mucoralis* was additionally performed in selected cases if deemed clinically appropriate, but not on a regular basis, and was therefore not analyzed in this study. All detected viruses were assigned to be pathologic, and for the detection of HSV, the relevant viral load was defined as >50,000 copies per mL. Bacteria that are part of the normal oral and pharyngeal flora and those that could be detected in such a small number that a more precise specification was not possible were defined as non-pathogenic. Regarding the detection of fungi, yeasts and not otherwise specified Penicillium species were defined as non-pathogenic (refer to Figure 3).

### 2.5. Statistical Analysis

All statistical analyses and figures were performed and obtained using the Statistical Package for Social Sciences statistical software, version 27.0 (IBM Corp., Armonk, NY, USA) and GraphPad Prism, version 9 for macOS (GraphPad Software, La Jolla, CA, USA). Continuous values are presented as the median with interquartile range (IQR). Categorial variables are expressed as a number with percentages (%).

### 2.6. Endpoint

The primary objective of this study was to determine the rate of pathogenic microorganisms detected in BAL fluid that led to a change in the anti-infective treatment approach in AL patients who are already under broad-spectrum antibiotic and antifungal therapy.

## 3. Results

In total, 88 patients met the inclusion criteria. Fifty-nine patients (67%) were male, and the median age at FB was 59 years (IQR: 48–68). The underlying disease was AML in 76 patients (86%) and ALL in 12 patients (14%; for patient characteristics, refer to Table 1).

### 3.1. Diagnostic Assessment and Clinical Courses

Diagnostic FB with BAL was performed in 1 patient (1%) with newly diagnosed AL prior to the start of chemotherapy, in 51 patients (58%) during induction chemotherapy, in 7 patients (8%) during consolidation chemotherapy, in 21 patients (24%) during salvage-chemotherapy, and in 8 patients (9%) during non-intensive chemotherapy. At the time of BAL, profound neutropenia was present in 83 patients (94%) and high-risk neutropenia (defined as neutrophile count <500/µL over 7 days) in 38 (43%). The median duration of neutropenia was 15 days (IQR: 10–25) before FB. The median value of the C-reactive protein (CrP) on the day of BAL was 162 mg/L (IQR: 105–217). Fever was observed in 28 patients (32%), and 10 patients (11%) had subfebrile body temperature. In 25 patients (28%), LDCT revealed typical infiltrates of lobar pneumonia. Atypical infiltrates were found in 63 cases (72%). Invasive mechanical ventilation due to pneumonia was required in 17 patients (19%), and a total of 12 patients (14%) died from respiratory insufficiency within 30 days after the onset of pneumonia. None of the patients had bronchoscopy-associated complications. In particular, no bleeding events occurred during FB.

### 3.2. Microbiological Findings in BAL

At least one pathogen was identified that most likely caused the infection episode in 41 cases (47%, Figure 3). Pathogenic bacteria (one or more germs) were found in 2 patients (2%), viruses (one or more germs) in 13 (15%), and fungi (one or more germs) in 16 cases (18%). In ten patients (11%), a combination of pathogenic bacteria, viruses, and fungi was detected: In three patients (3%), all three pathogens were detected; in four patients (5%), there was concomitant infection with viruses and fungi; in two patients (2%) there was bacteria and fungi; and in one patient (1%) bacteria and viruses occurred. For a more detailed presentation of the distribution of germs and whether the germs were classified as pathogenic or not, refer to Figure 3a–c. The most often detected pathogens were as follows: For bacteria, *Stenotrophomonas maltophilia* (*n* = 4; 40% of all pathogenic bacteria); for viruses, *Herpes simplex virus 1* (*n* = 9; 38% of all pathogenic viruses); and for fungi, *Aspergillus fumigatus* (*n* = 25; 97% of all pathogenic fungi). None of the detected bacterial strains were identified as a strain with extended or multi-resistance against anti-infective compounds.

### 3.3. Impact of BAL on Anti-Infective Treatment

All patients were treated with broad-spectrum antibiotic and antifungal agents according to hospital-based standard operation procedures for patients with FN (Table 1 and Figure 2). Seventy-three patients (83%) received meropenem, fourteen patients received ceftazidim (16%), and one patient was treated with piperacillin/tazobactam (1%) as broad-spectrum antibiotic therapy. In all patients, additional agents for the coverage of most Gram-positive microorganisms were administered: 64 (73%) patients were treated with vancomycin or linezolid, and additional extended therapy with other antibiotics such as tigecycline, gentamicin, daptomycin, and fosfomycin was administered in 24 patients (27%). One patient was additionally treated with metronidazole due to *Clostridium difficile* intestine infection. For antifungal treatment, in the majority, sixty-eight patients (77%), amphotericin B was administered, whereas nine patients (10%) were treated with caspofungin, seven patients (8%) were treated with voriconazole, three patients (3%) with posaconazole, and one patient (1%) with anidulafungin. Different therapeutic approaches were used due to potential interactions with agents to treat AML or ALL, other contraindications, or patients’ intolerance.

In every patient, prophylactic antiviral therapy with aciclovir and prophylaxis against *P. jirovecii* was administered irrespective of the occurrence of FN. Two patients (2%) received additional therapy with oseltamivir due to previously laboratory-confirmed influenza virus infection (for detailed information about anti-infective agents, refer to Table 1).

In 74 patients (84%), FB with BAL assessment did not lead to any therapeutic consequences because no pathogen was detected that was not covered by empiric anti-infective treatment. Regarding viral infections, treatment change could be observed in nine (10%) patients diagnosed with *Herpes simplex 1* pneumonia and the relevant viral load (>50,000 copies) by changing aciclovir dosage. In five patients (5%), *Influenza A or B virus* was detected and led to therapeutic consequences, with the administration of oseltamivir in three patients, whereas the remaining two patients were already under oseltamivir treatment due to positive nasopharyngeal swabs prior to BAL. Regarding the detection of fungi, a therapeutic consequence was observed in one patient (1%) in whom *Pneumocystis* spp. Were detected in BAL. This patient received cotrimoxazole in the therapeutic dosage (Table 2). Of note, the detected germ was not *P. jirovecii*. Therapeutic consequences against bacteria occurred in one patient with the detection of *Mycoplasma pneumoniae* in BAL fluid. This patient additionally received azithromycin as a specific anti-infective agent. All other bacterial isolates (*S. maltophilia* included) were covered by calculated antimicrobial therapies already administered in this patient cohort. None of the detected bacterial strains had multi-drug resistance. Negative microbiological BAL fluid did not result in de-escalation or withdrawal of any empiric anti-infective treatment in accordance with our local standard operating procedures regarding FN in patients with hematological malignancies.

## 4. Discussion

Despite all the advances that have been made in the prevention and treatment of FN, AL patients still display the highest FN-related in-hospital mortality among all cancer patients, mainly due to a prolonged period of profound neutropenia [1,2,3,4,5]. However, even with extensive and repeated microbiological work-up, the detection of a pathogenic germ often remains unsuccessful, making targeted anti-infective therapies more difficult [4,7,8]. According to the current ESMO clinical practice guidelines on the management of FN, AML patients during induction therapy are particularly at risk for invasive fungal infections, and FB with BAL is recommended if any pulmonary infiltrate is detected by chest CT [1]. Nevertheless, the question of the utility of BAL to determine the causative agent of pulmonary infiltrates in patients with high-risk neutropenia and AL has been raised for decades [16,17,18,19]. In particular, the utility of FB and BAL under broad-spectrum antibiotics and antifungal treatment remains unclear. By investigating 88 BAL fluids of AL patients with clinically suspected infection and radiological infiltrates under broad-spectrum antibiotic and antifungal treatment, positive microbiological BAL fluids with clinically relevant pathogens were obtained in 47% of our cases. These results are consistent with previously reported studies describing 39–54% of positive BAL fluids [13,20,21,22,23]. However, in some studies, lower rates, with a range of approximately 23–32% of positive BAL fluids, were reported [12,14,24,25]. A long duration of profound neutropenia with a median time of 15 days due to AL in our patient cohort may explain this finding since the acquisition of different pathogens causing severe infections could be increasingly observed during long-lasting neutropenia [1].

Of note, despite the extensive empiric anti-infective treatment in our study population, in nearly every second BAL fluid (47%), relevant pathogens could be isolated. *Aspergillus fumigatus* was the most detected pathogen albeit the empiric anti-fungal treatment in every patient, indicating the high risk of invasive aspergillosis in patients with AL underlying previous reported data [20,22,25]. Remarkably, no multi-resistant pathogens were isolated despite the administration of broad-spectrum antibiotics and anti-fungal agents. One of the few prospective trials investigating BAL-driven antimicrobial treatment in patients with hematological malignancies and detectable lung infiltrates is a multi-center study by the SEIFEM (SORVEGLIANZA EPIDEMIOLOGICA INFEZIONI NELLE EMOPATIE) group [26]. Marchesi et al. reported the detection of putative causal agents in 111 cases (76%), which provided guidance for antimicrobial treatment in 89 cases (61%). The authors reported considerably high rates of detected pathogens in BAL fluid and BAL-guided treatment modifications compared to our results. Possible reasons for these differences could be explained by different inclusion criteria since not all patients have been pre-treated with empiric antibiotics at the time of lung infiltration detection in the study population reported by Marchesi and colleagues (69–74% versus 100% in our study population) [26]. Moreover, the authors did not provide further information on the definition of broad-spectrum antimicrobial therapy, which likely differs from our inclusion criteria. Additionally, only 5–8% of patients reported by Marchesi et al. were treated with empirical antifungal therapy at the time of lung infiltrate detection in contrast to 100% in our study population [26].

BAL findings resulted in subsequent anti-infective treatment changes in 15% of patients, which is in line with one of the very few studies investigating the impact of BAL in patients with AL under extensive anti-infective therapies, showing a low rate of 9.3% of BAL-guided treatment changes [12]. In all other reported patient cohorts, the rate of treatment changes was higher with a range of 24–57% [13,14,20,21,22,23,24,25]. This could be mainly explained by the more frequent use of pre-emptive treatment approaches in the other reported studies, whereas here we report on patients who were exclusively treated empirically with broad-spectrum antibiotic and combined antifungal therapies. This approach is the standard of care in our department because many patients respond to empiric anti-infective treatment approaches and do not have to undergo FB or LDCT. Additionally, due to the residual risk of false-negative BAL fluids and a possible delay of anti-infective treatment, there is a consensus on empiric treatment approaches in AL patients with suspected infections in our department.

Since most treatment changes in our patient cohort were required due to the detection of *Herpes simplex virus 1* or *Influenza A/B virus*, nasopharyngeal swabs, throat rinses, and/or sputum might be a reasonable method prior to BAL to detect herpes and influenza viruses. By excluding these cases in which pathogens might be detected non-invasively, only in 2 out of 88 patients (2%) were BAL-guided therapy changes observed. Even if a primary impact on the anti-infective treatment approach in these patients is seen in a minority in our cohort, there are some aspects that should be discussed when considering the necessity of FB with BAL: Leukemia patients undergo several therapy cycles, and the majority of patients are allocated to allogeneic stem cell transplantation for consolidation therapy. The isolation of pathogens from BAL or detection of fungal pneumonia in LDCT during infection periods in previous cycles of chemotherapy might lead to different prophylactic approaches when a patient undergoes, for example, the second induction chemotherapy or an allogeneic stem cell transplantation.

Although there are already several publications on the utility of BAL in hematological patients with and without FN, we herein investigated one of the few diagnostic yields of BAL in a large, homogeneous collective consisting only of patients with AL and subsequent prolonged and profound neutropenia. In addition, all patients included in this study were treated with broad-spectrum empiric anti-infective therapies and consistent anti-infective prophylaxis leading to a minimization of a study population selection bias. Furthermore, patients who previously underwent allogeneic stem cell transplantation were excluded. Moreover, in all patients, FB with BAL was based on the most recent LDCT findings to increase the possible utility of BAL. Nevertheless, the question remains unanswered as to why, in 53% of patients with radiological infiltrates, no pathogens could be detected in LDCT-finding conducted BAL. Compared to other studies in non-hematological patients, where BAL is used for the confirmation of diagnosis (infection or malignancy), the rate of negative BAL results is high in our cohort [27]. This might be explained by the empirically administered anti-infective agents previous to BAL, resulting in a decrease in bacterial, viral, or fungal load in the respiratory tract.

Due to the retrospective study design, there is a risk of potential selection bias and residual confounding factors in this analysis, particularly due to a missing control group. Moreover, even though we did not observe any multi-resistant bacteria, our observations cannot thoroughly be transferred to other European or non-European countries based on different local resistances. Furthermore, microbiological testing was limited to frequent opportunistic fungi and viruses and did not regularly include testing for *Mucor mucoralis*. Further large prospective studies are necessary for this patient cohort, comparing BAL-guided anti-infective treatment versus empiric anti-infective treatment, to establish predicting factors for BAL-guided treatment changes.

## 5. Conclusions

Our results indicate that despite broad-spectrum anti-infective treatment approaches, in approximately half of all patients with AL and FN and/or increased CrP levels and radiologically confirmed diagnosis of pneumonia, pathogens could still be isolated in BAL samples. Nevertheless, the detection of pathogens did not lead to frequent changes in anti-infective treatments, with most changes performed in patients with herpes simplex and influenza virus detection. Although no FB-related complications occurred in our study population, these pathogens might also be detected in less invasive examinations. In conclusion, the need for FB with BAL in patients with AL who are already receiving broad-spectrum empiric anti-infective treatment should be weighed carefully.

## Figures and Tables

**Table 1:** Overview of patient characteristics and anti-infective treatment.

Total Cohort (*n* = 88)	
**Median age in years (IQR)**	59 (48–68)
**Male Sex, *n* (%)**	59 (67)
**Underlying disease, *n* (%)**	
Acute myleoid leukemia	76 (86)
Acute lymphoblastic leukemia	12 (14)
**Time point of FB with BAL, *n* (%):**	
Prior any systemic oncological therapy	1 (1)
During induction cycles	51 (58)
During consolidation cycles	7 (8)
During salvage chemotherapy	21 (24)
During low-dose chemotherapy	8 (9)
**Aplasia at FB with BAL, *n* (%):**	83 (94)
Duration of aplasia, days (IQR)	15 (10–25)
High-risk neutropenia	38 (43)
**Fever at FB with BAL, *n* (%)**	28 (32)
**Chest computed tomography, *n* (%):**	88 (100)
Lobar pneumonia	25 (28)
Atypical pneumonia	63 (72)
**Invasive ventilation <30 days after the onset of pneumonia, *n* (%)**	17 (19)
**Pneumonia-related death <30 days after the onset of pneumonia, *n* (%)**	12 (14)
**Anti-infective treatment against bacteria at FB, *n* (%)**	88 (100)
Meropenem	73 (83
Ceftazidim	14 (16)
Piperacillin/Tazobactam	1 (1)
Vancomycin or Linezolid	64 (73)
Tigecycline	22 (25)
Fosfomycin	4 (5)
Gentamicin	3 (3)
Daptomycin	1 (1)
**Anti-infective treatment against fungal infection at FB, *n***	88 (100)
Amphotericin B	68 (77)
Caspofungin	9 (10)
Voriconazole	7 (8)
Posaconazole	3 (4)
Anidulafungin	1 (1)
Prophylaxis against Pneumocystis jirovecii (Cotrimoxazole *n* = 89, Pentacarinate *n* = 2)	88 (100)
**Anti-infective treatment against viruses at FB, *n* (%)**	88 (100)
Aciclovir prophylaxis	88 (100)
Oseltamivir	2 (2)

IQR: Interquartile range; FB: Flexible bronchoscopy; BAL: Bronchoalveolar lavage; ARDS: Acute respiratory distress syndrome.

**Table 2:** BAL-driven changes of anti-infective treatment approaches.

	Pathogen Detected in BAL	Prior Anti-Infective Treatment to BAL *	Changes in Anti-Infective Treatment after BAL
**Case 1**	*Influenza A*	Meropenem, vancomycin, amphotericin B	Oseltamivir
**Case 2**	*Herpes simplex virus 1* **	Meropenem, vancomycin, amphotericin B	Adaption of aciclovir dosage
**Case 3**	*Herpes simplex virus 1* **	Meropenem, vancomycin, amphotericin B	Adaption of aciclovir dosage
**Case 4**	*Herpes simplex virus 1* **	Meropenem, vancomycin, caspofungine	Adaption of aciclovir dosage
**Case 5**	*Influenza A*	Meropenem, tigecycline, amphotericin B	Oseltamivir
**Case 6**	*Influenza A*	Ceftazidim, tigecycline, amphotericin B	Oseltamivir
**Case 7**	*Herpes simplex virus 1* **	Ceftazidim, vancomycin, amphotericin B	Adaption of aciclovir dosage
**Case 8**	*Herpes simplex virus 1* **	Meropenem, tigecycline, amphotericin B	Adaption of aciclovir dosage
**Case 9**	*Herpes simplex virus 1* **	Meropenem, vancomycin, amphotericin B	Adaption of aciclovir dosage
**Case 10**	*Herpes simplex virus 1* ** and *mycoplasma pneumoniae*	Meropenem, linezolid, amphotericin B	Azithromycine and adaption of aciclovir dosage
**Case 11**	*Herpes simplex virus 1* **	Meropenem, tigecycline, voriconazole	Adaption of aciclovir dosage
**Case 12**	*Herpes simplex virus 1* ** and *pneumocystis* spp.	Meropenem, tigecycline, amphotericin B	Adaption of aciclovir and cotrimoxazole dosage

* Including aciclovir and cotrimoxazole prophylaxis in all 12 patients. ** Detection of >50,000 copies; BAL: Bronchoalveolar lavage.

## Data Availability

The datasets generated during and/or analyzed during the current study are available from the corresponding author on reasonable request.
